# Tumeur phyllode chez une jeune adolescente de 12 ans: à propos d’un cas et revue de la littérature

**DOI:** 10.11604/pamj.2016.25.20.10219

**Published:** 2016-09-26

**Authors:** Karima Issara, Majdouline Houjami, Souha Sahraoui, Zineb Bouchbika, Nadia Benchakroun, Hassan Jouhadi, Nezha Tawfiq, Abdellatif Benider

**Affiliations:** 1 Centre Mohammed VI de traitement des cancers, CHU Ibn Rochd Faculté de médecine et pharmacie, Université Hassan II, Casablanca, Maroc

**Keywords:** Tumeur phyllode, sein, enfant, traitement, radiothérapie, Phyllodes tumor, breast, child, treatment, radiotherapy

## Abstract

Les tumeurs phyllodes du sein sont des tumeurs très rares et restent exceptionnelles chez les enfants et les adolescents, leurs traitement est basé sur la chirurgie et la radiothérapie, avec un bon pronostic. Nous rapportons le cas d'une adolescente âgée de 12 ans, qui s'est présentée pour une masse du sein gauche. Le diagnoctic d'une tumeur phyllode a été retenu après bilan et histologie. Le traitement a consisté en une tumorectomie large sans traitement adjuvant ; avec une bonne évolution à un recul de deux ans.

## Abstract (en)

Phyllodes tumors of the breast are very rare and are exceptional in children and adolescents; their treatment is based on surgery and radiotherapy, with a good prognosis. We report the case of a 12-year old teenage girl presenting with a mass in the left breast. The diagnosis of a phyllodes tumor was confirmed on the basis of clinical, imaging and histological examinations. Treatment consisted of a large tumorectomy without adjuvant therapy, with good evolution during a 2-year follow-up.

## Introduction

Les tumeurs phyllodes du sein sont des tumeurs très rares, représentant uniquement 1% de toutes les tumeurs du sein. Elles sont plus fréquentes chez la femme en activité génitale que chez la femme ménopausée. (Pic de fréquence 35-55 ans) Ces tumeurs ont été décrites pour la première fois en 1893 par Johannes Muller, le nom de ces tumeurs est dérivé du mot grec phyllon et Eidos qui signifie feuille vu que la tumeur prend la forme d'une feuille. Elles sont des tumeurs fibroépithéliales, proches des fibro adénomes qui représentent le principal diagnostic différentiel. Elles sont le plus souvent bénignes (60 à 70%) et elles sont considérées comme un groupe distinct de néoplasies rares : 0.3 à 1% des néoplasies mammaires.

## Patient et observation

**Figure 1:**
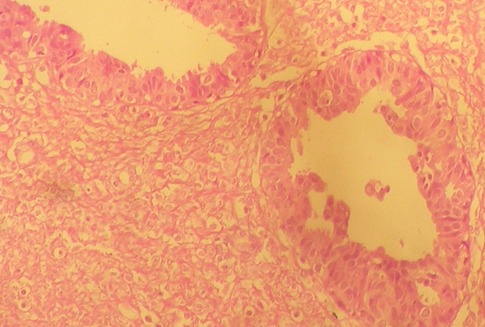
Aspect histologique d’une tumeur phyllode de grade 2

**Figure 2:**
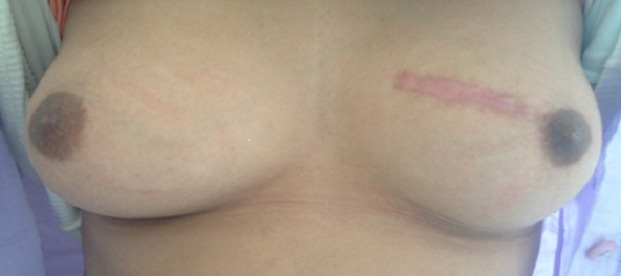
Aspect clinique post chirurgical (première tumorectomie)

Nous rapportons l'observation d'une adolescente âgée de 12 ans, réglée depuis l'âge de 11 ans, sans antécédents pathologiques particuliers personnels ou familiaux, qui consulte pour une masse du sein gauche. Le début de la symptomatologie remonte à 8 mois auparavant par l'autopalpation d'une masse ferme indolore au niveau du sein gauche, augmentant progressivement de volume, ce qui a motivé une consultation. L'examen clinique des seins a trouvé un nodule au niveau du quadrant supéro interne du sein gauche, il s'agit d'un nodule de 5 cm de grand axe, de consistance ferme, indolore, mobile par rapport au plan cutané et au plan profond, sans signes inflammatoires ou lésions cutanés en regard. L'examen des aires ganglionnaires ne retrouve pas d'adénopathies axillaires ou sus claviculaires. Le reste de l'examen somatique ne retrouve pas d'anomalie ailleurs. Une échographie mammaire a été réalisée objectivant un nodule hypo-échogène, mesurant 5 cm, de contours réguliers ; le diagnostic d'un adénofibrome juvénile a été alors retenu. La patiente a bénéficié d'une tumorectomie dont l'analyse anatomopathologique (Figure 1) a conclu en une tumeur phyllode de grade 2, avec des limites périphériques tumorales par endroits. L'examen post opératoire des seins objective des seins légèrement asymétriques, avec présence d'une cicatrice de tumorectomie sans masse palpable (Figure 2). Le dossier a été discuté en réunion pluridisciplinaire vu les limites tumorales, et la décision d'une tumorectomie large a été posée vu le développement suffisant des seins permettant la conservation. L'anatomopathologie de la reprise chirurgicale a objectivée la présence de lésions de mastopathie scléro-kystique non proliférante, sans aucune prolifération tumorale. Aucun traitement adjuvant n'est indiqué dans ce cas. La patiente est toujours en rémission complète à 2 ans de recul actuellement.

## Discussion

Cette étude de cas est une présentation d'une tumeur phyllode maligne survenant chez une adolescente de 12 ans. Les tumeurs phyllodes représentent uniquement 0.3 - 1% des tumeurs mammaires, elles sont plus fréquents chez la jeune femme et restent très rares à un si jeune âge [1, 2] (Tableau 1). Sur le plan clinique, la tumeur phyllode est une tumeur de croissance rapide entrainant une augmentation du volume mammaire assez caractéristique ; elle se manifeste par une masse volumineuse, unilatérale, ferme et plus au moins élastique. Des modifications cutanées type érythème [3, 4], vergeture ou signes inflammatoires sont présentes uniquement en cas de tumeur de taille importante ou de siège superficiel. Les tumeurs phyllodes posent un problème de diagnostic différentiel avec les fibroadénomes [1, 3]. Sur le plan radiologique; La mammographie retrouve les critères sémiologiques d'une masse typiquement bénigne comme le fibroadénome à savoir une forme ronde, des contours réguliers et une densité élevée [5]. L'échographie peut mettre en évidence des plages d'échostructure hétérogène avec des zones anéchogènes kystiques [2–4]. L'IRM retrouve également les critères sémiologiques d'une tumeur bénigne, ainsi elle ne permet pas de faire le diagnostic différentiel entre la tumeur phyllode et l'adénofibrome [3, 4]. Histologiquement la tumeur phyllode est une tumeur fibroépithéliale avec présence d'une hyperplasie stromale. Le grade histopronostique est établi sur l'associasion des facteurs histologiques péjoratifs notamment le nombre de mitoses par 10 champs, la sévérité des atypies cellulaires, l'interface tumeur/parenchyme sain, la présence de nécrose tumorale et la densité stromale [4]. La tumeur est classée en fonction du grade histopronostique en 3 grades ; le grade 1correspond à une tumeur bénigne sans facteurs histologique péjoratifs et aucun risque de récidive, le grade 2 correspond à une tumeur borderline avec présence d'au moins un facteur péjoratif et le grade 3 correspond à un sarcome phyllode avec au moins 3 facteurs péjoratifs et un risque de métastases évalué à 25% à 3 ans [6]. Sur le plan thérapeutique, la chirurgie représente le traitement standard. Une tumorectomie élargie avec une marge de sécurité de 10 mm est indiquée pour les tumeurs de grade 1 et 2 [6]. Et une mastectomie simple sans curage ganglionnaire est indiquée pour les tumeurs de grade 3, ou pour les tumeurs de plus de 5 cm [3–5]. La radiothérapie adjuvante trouve sa place dans le cas d'une tumeur de grade 3, une 3ème récidive locale, ou une récidive après une mastectomie. Les tumeurs phyllodes se comportent relativement de façon bénigne, cependant on peut avoir une récidive locale dans 25% des cas [4, 5] et en particulier après un traitement conservateur.

**Tableau 1:** Tableau récapitulatif des cas signalés dans la littérature

	age	clinical appearance	Time limit	size	echo appearance	histology	Treatment	evolution	Recoil
**our study**	12	Left breast mass	8 months	5 cm	Hypoechoic nodule smooth edges	Phyllodes tumor grade 2	lumpectomy	Favorable	2 years
**Martino 2001 Italie**	13	Right breast mass	3 months	6cm	Mass under inhomogeneous regular edges areal	Phyllodes low grade tumor	lumpectomy	Favorable	15 months
**Pistolese 2009 Italie**	11	flow nipple	1 months	4 cm	Multiple hypoechoic solid lesions	Phyllodes tumor BPD	lumpectomy	Favorable	1 year
**Sorelli 2010 United Kingdom**	11	Painful swollen right breast	3 weeks	9 cm	Hypoechoic nodule	Phyllodes tumor grade2	lumpectomy	Favorable	3 years
**Graciela 2010 Espagne**	11	Right breast mass	1 months	6 cm	Hypoechoic nodule, no calcifications	Phyllodes low grade tumor	lumpectomy	Relapse three weeks post op: Patey	2 years

## Conclusion

Les tumeurs phyllodes de l'enfant et l'adolescent sont des tumeurs très rares. Notre observation illustre les difficultés de diagnostic différentiel de ce type de tumeurs dont le traitement de base à l'heure actuelle est dominé par la chirurgie. **Consentement:** le consentement éclairé écrit a été obtenu de la patiente pour la publication de ce rapport de cas et toutes les images qui les accompagnent. Une copie du consentement écrit est disponible pour l´examen par le rédacteur en chef de ce journal.
